# A small-molecule inhibitor suppresses the tumor-associated mitochondrial NAD(P)^+^-dependent malic enzyme (ME2) and induces cellular senescence

**DOI:** 10.18632/oncotarget.3907

**Published:** 2015-05-19

**Authors:** Ju-Yi Hsieh, Shao-Yu Li, Wen-Chen Tsai, Jyung-Hurng Liu, Chih-Li Lin, Guang-Yaw Liu, Hui-Chih Hung

**Affiliations:** ^1^ Department of Life Sciences, National Chung Hsing University, Taichung, Taiwan; ^2^ Institute of Microbiology & Immunology, Chung Shan Medical University, and Division of Allergy, Immunology, and Rheumatology, Chung Shan Medical University Hospital, Taichung, Taiwan; ^3^ Institute of Genomics and Bioinformatics, National Chung Hsing University, Taichung, Taiwan; ^4^ Agricultural Biotechnology Center (ABC), National Chung Hsing University, Taichung, Taiwan; ^5^ Institute of Medicine, Chung Shan Medical University, Taichung, Taiwan

**Keywords:** allosteric inhibitor, selective inhibitor, non-competitive inhibition, mutagenesis analysis, cellular senescence

## Abstract

Here, we found a natural compound, embonic acid (EA), that can specifically inhibit the enzymatic activity of mitochondrial NAD(P)^+^-dependent malic enzyme (m-NAD(P)-ME, ME2) either *in vitro* or *in vivo*. The *in vitro* IC_50_ value of EA for m-NAD(P)-ME was 1.4 ± 0.4 μM. Mutagenesis and binding studies revealed that the putative binding site of EA on m-NAD(P)-ME is located at the fumarate binding site or at the dimer interface near the site. Inhibition studies reveal that EA displayed a non-competitive inhibition pattern, which demonstrated that the binding site of EA was distinct from the active site of the enzyme. Therefore, EA is thought to be an allosteric inhibitor of m-NAD(P)-ME. Both EA treatment and knockdown of m-NAD(P)-ME by shRNA inhibited the growth of H1299 cancer cells. The protein expression and mRNA synthesis of m-NAD(P)-ME in H1299 cells were not influenced by EA, suggesting that the EA-inhibited H1299 cell growth occurs through the suppression of *in vivo* m-NAD(P)-ME activity EA treatment further induced the cellular senescence of H1299 cells. However, down-regulation of the enzyme-induced cellular senescence was not through p53. Therefore, the EA-evoked senescence of H1299 cells may occur directly through the inhibition of ME2 or a p53-independent pathway.

## INTRODUCTION

Altered cellular metabolism is a hallmark of cancer. Targeting human malic enzymes could be an effective approach to inhibit tumor growth [1]. Malic enzymes (MEs) are a family of homotetrameric enzymes that catalyze the reversible oxidative decarboxylation of L-malate to pyruvate, with a simultaneous reduction of NAD(P)^+^ to NAD(P)H. The ME family is broadly distributed throughout nature and plays important roles in the metabolic pathways of organisms. In mammals, the enzyme can be found as three isoforms, which are defined by their subcellular localization and cofactor specificity. Both the cytosolic and mitochondrial NADP^+^-dependent malic enzymes (c-NADP-ME, ME1; m-NADP-ME, ME3, respectively) utilize NADP^+^ as a cofactor and play important roles in lipogenesis by providing NADPH for the biosynthesis of long-chain fatty acids and steroids [2, 3]. Therefore, c-NADP-ME, together with acetyl-CoA carboxylase, fatty acid synthase and glucose-6-phosphate dehydrogenase, is classified as a lipogenic enzyme [2, 4].

m-NAD(P)-ME (ME2), which is distinctive from the other two mammalian isoforms, has a dual cofactor specificity and a complex allosteric regulatory system that controls its catalytic activity. It can utilize either NAD^+^ or NADP^+^ as a cofactor and displays a cooperative behavior with respect to its substrate L-malate. Additionally, its enzymatic activity can be allosterically activated by fumarate [5] and inhibited by ATP [6–9]. The allosteric properties of m-NAD(P)-ME imply its specific role in malate and glutamine oxidation in tumor mitochondria [7–10].

Although m-NAD(P)-ME may preferentially use NAD^+^ under physiological conditions, the enzyme can generate NADH and NADPH in the mitochondria and, thus, may play dual roles in energy production and reductive biosynthesis [11]. Cancer cells must encounter the demands of bioenergetics and biosynthesis for growth and proliferation. Therefore, m-NAD(P)-ME is considered a possible molecular target for cancer drug discovery. By producing NADH and pyruvate, the m-NAD(P)-ME isoform may play an important role in energy production in rapidly proliferating tissues and tumor cells [6, 12, 13]. By producing NADPH, the enzyme generates the reducing equivalents for lipid biosynthesis and glutathione reduction [11]. In tumor cells, glutamine and glutamate, not glucose, are the major energy sources [4, 13, 14], and m-NAD(P)-ME may play an important role in glutaminolysis, the metabolism of glutamine, for energy production in rapidly proliferating cells [10, 11, 13, 15, 16]. The oxidation of glutamine via the tricarboxylate cycle and the entry and oxidation of malate via m-NAD(P)-ME in tumor cells might be regulated in a coordinated manner [7, 14, 15]. Recently, an elegant study demonstrated that the c-NADP-ME and m-NAD(P)-ME enzymes are involved in senescence and are closely linked to p53. A reciprocal regulatory relationship exists between p53 and malic enzymes and governs the irreversible fate of the cell, and this regulation is mediated by the malic enzyme-involved metabolic pathway [17, 18]. Additionally, the knockdown of m-NAD(P)-ME can lead to the induction of erythroid differentiation in human erythroleukemia cells [19], and the knockdown of m-NAD(P)-ME or the administration of dimethyl-malate (substrate analog of m-NAD(P)-ME) inhibits tumor growth *in vivo* [20].

We have highlighted the roles of m-NAD(P)-ME in cutaneous melanoma [21]. m-NAD(P)-ME mRNA and protein expression significantly increased during melanoma progression. Additionally, m-NAD(P)-ME knockdown attenuated melanoma cell proliferation *in vitro*. m-NAD(P)-ME depletion in A375 melanoma cells (wild-type p53) enhanced AMPK activity, increased p53 levels, and up-regulated the p53 downstream target p21, which inhibits the cell cycle. Additionally, m-NAD(P)-ME ablation resulted in reduced cellular ATP levels and elevated cellular ROS production, which activated the AMP-activated protein kinase (AMPK) pathway and inhibited acetyl-CoA carboxylase (ACC) [21].

The most striking difference among the human ME isoforms is that c-NADP-ME and m-NADP-ME are non-allosteric enzymes, but m-NAD(P)-ME can be allosterically activated by fumarate. Crystal structures of human m-NAD(P)-ME in complex with their organic ligands provided evidence that two extra ligand binding sites are present in each monomer of the enzyme isoform [22–24]. One site is located at the dimer interface and binds the allosteric activator, fumarate, and the other site is located at the tetramer interface and can bind another NAD or ATP molecule; this second binding site is named the “exo site” [23, 25].

In the fumarate binding pocket, the carboxylate group of fumarate is ion-paired with the guanidinium group of Arg67 and Arg91. Mutagenesis studies have shown that these Arg residues are exclusively required for the activating effect of fumarate [20]. In addition, the anionic amino acid residue Glu59, which is ion-paired with Arg67, has a profound effect on the fumarate activating effect [23, 26]. Another anionic residue, Asp102, has also been shown to have a remarkable effect on fumarate activation [27]. In the exo site, the NAD^+^ molecule interacts with Arg197, Arg542 and Arg556 [24]. These residues contribute to the binding of the nucleotide and to the quaternary structure stability [28].

**Figure 1:**
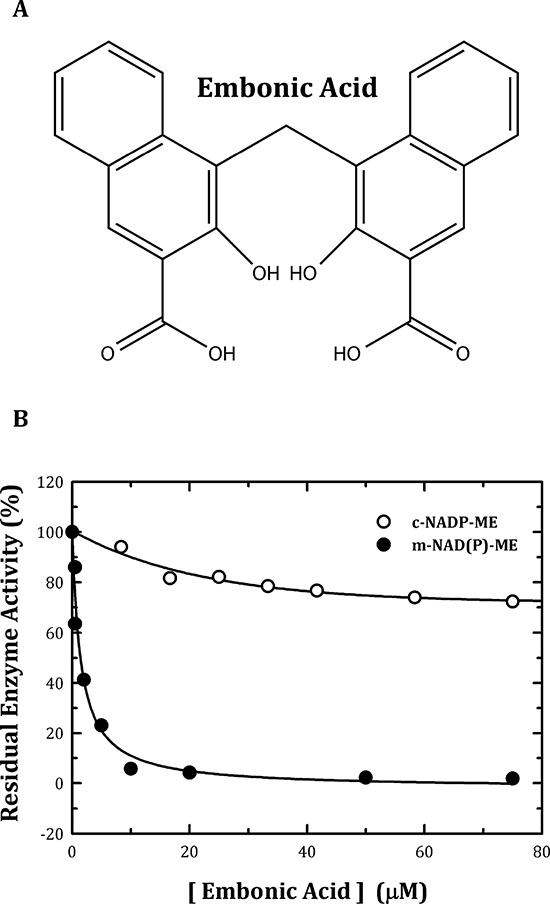
Chemical structure of embonic acid (EA) and inhibitory effect of EA on human m-NAD(P)-ME and c-NADP-ME **A.** Chemical structure of EA. **B.** The assay mixture contained 40 mM malate, 10 mM MgCl_2_, 2 mM NAD^+^ or NADP^+^ and 50 mM Tris-HCl (pH 7.5) with various concentrations of EA. The concentrations of EA ranged from 0 to 75 μM. Closed circles, m-NAD(P)-ME; open circles, c-NADP-ME.

A huge amount of biochemical and biophysical data have been generated in long-term studies of ME, and from these studies, the catalytic mechanism of this enzyme family, including a number of issues related to the isoform-specific allosteric regulation, the cooperative mechanism and the subunit-subunit interactions, have become clearer [29–35]. In this paper, we report a chemical compound, embonic acid (EA) (Figure 1), that can specifically inhibit m-NAD(P)-ME, rather than c-NADP-ME. The IC_50_ value of EA for m-NAD(P)-ME was approximately 1 μM, which is smaller than some preclinical drugs. EA is thought to be an allosteric inhibitor specific to m-NAD(P)-ME as mutagenesis analysis revealed that the possible binding site for EA is located at the fumarate binding site or at the dimer interface near the site. Additionally, EA inhibits the growth of H1299 cancer cells and then induces cellular senescence through the suppression of *in vivo* m-NAD(P)-ME activity. In clinical implication, the small-molecule inhibitor of m-NAD(P)-ME, embonic acid, may be taken advantage for cancer therapy.

## RESULTS AND DISCUSSION

### Isoform-specific inhibitor of human m-NAD(P)-ME

By *in vitro* testing of a chemical compound library, we found a natural chemical, EA, which can inhibit m-NAD(P)-ME much more potently than c-NADP-ME (Figure 1B, closed and open circles, respectively). The *in vitro* IC_50_ value of EA for m-NAD(P)-ME was 1.4 ± 0.4 μM (Table 1). Because c-NADP-ME was much less sensitive to inhibition by EA, this compound could be an isoform-specific inhibitor that can distinguish m-NAD(P)-ME from c-NADP-ME.

**Table 1:** IC_50_ values of embonic acid against the WT and mutant m-NAD(P)-ME enzymes

Characteristics	m-NAD(P)-ME	Embonic acid
	WT	1.4 ± 0.4 μM
*Tetramer interface mutant*	H142A/D568A	2.1 ± 0.1 μM
*Exo site mutant(at tetramer interface)*	R197E	2.8 ± 0.1 μM
	R542V	1.5 ± 0.2 μM
*Dimer interface mutant*	Q51A/E90A	29.5 ± 7.4 μM
*Fumarate site mutant(at dimer interface)*	E59N	5.1 ± 0.7 μM
	R67A	>75 μM
	R91A	>75 μM
	R67A/R91A	>75 μM
	K57S/E59N/K73E/D102S	>75 μM

### Inhibitory effect of embonic acid (EA) on the dimer interface, tetramer interface, exo site and fumarate site mutants of human m-NAD(P)-ME

The binding site of EA could possibly reside at the dimer interface, tetramer interface, exo site or fumarate binding site. To address this question, the following mutants of human m-NAD(P)-ME were created to interrupt the dimer and tetramer interfaces, as well as the exo site and fumarate binding site. The Q51A/E90A and H142A/D568A enzymes represented the dimer interface and tetramer interface mutants, respectively [29]; the R197E and R542V represented the exo site mutants [23, 28]; and E59N, R67A, R91A, R67A/R91A and K57S/E59N/K73E/D102S represented the fumarate binding site mutants [23, 26, 27, 31, 36]. It is worth noting that the exo site is located at the tetramer interface and the fumarate binding site is at the dimer interface; therefore, the dimer interface mutant Q51A/E90A may not bind fumarate. Similarly, the H142A/D568A tetramer interface mutant may not have a functional exo site and not bind ligand.

**Figure 2:**
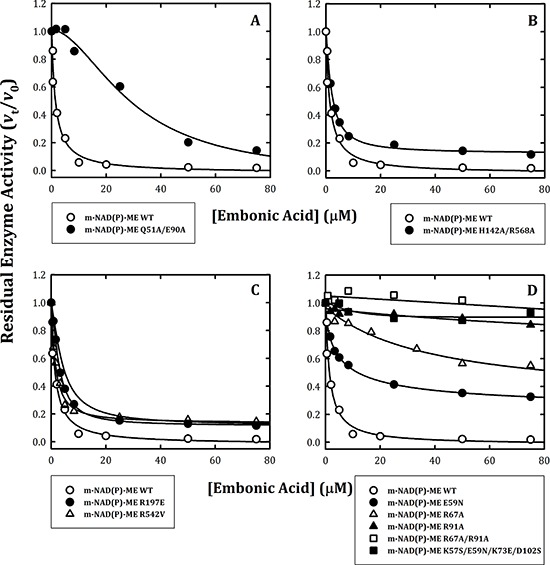
Inhibitory effects of embonic acid (EA) on wild-type and mutant m-NAD(P)-ME The assay mixture contained 40 mM malate, 10 mM MgCl_2_, 2 mM NAD^+^ and 50 mM Tris-HCl (pH 7.5) with various concentrations of EA that ranged from 0 to 75 μM. **A.** m-NAD(P)-ME WT and the Q51A/E90A dimer interface mutant; **B.** m-NAD(P)-ME WT and the H142A/D568A tetramer interface mutant; **C.** m-NAD(P)-ME WT and the R197E and R542V exo site mutants; **D.** m-NAD(P)-ME WT and the E59N, R67A, R91A, R67A/R91A and K57S/E59N/K73E/D102S fumarate site mutants.

For the dimer interface mutant Q51A/E90A, EA inhibited this enzyme less than the WT enzyme (Figure 2A), with an IC_50_ value of 29.5 ± 7.4 μM compared to the IC_50_ value of 1.4 ± 0.4 μM for WT. In contrast, the tetramer interface mutant H142A/D568A and the exo site mutants R197E and R542V were inhibited at a level similar to that of the WT enzyme by EA (Figure 2, 2B and 2C, respectively) with IC_50_ values of 2.1 ± 0.1, 2.8 ± 0.1 and 1.5 ± 0.2 μM, respectively (Table 1). For the fumarate binding site mutants, the E59N enzyme was inhibited by EA with a slightly increased IC_50_ value of 5.1 ± 0.7 μM (Table 1; Figure 2D, closed circles). However, the R67A enzyme was less sensitive to EA inhibition (Figure 2D, open triangles), and the R91A and R67A/R91A mutants (Figure 2D, closed triangles and open squares, respectively) were almost insensitive to EA inhibition; their IC_50_ values were more than 75 μM. Furthermore, the K57S/E59N/K73E/D102S enzyme was also resistant to inhibition by EA (Figure 2D, closed squares). These data suggested that the inhibitory effect of EA on the enzyme did not occur through binding to the active site or to the exo site and that the putative binding site of EA is located at the fumarate binding site or at the dimer interface near the site.

### Ability of fumarate to reverse the inhibition of m-NAD(P)-ME activity by EA

**Figure 3:**
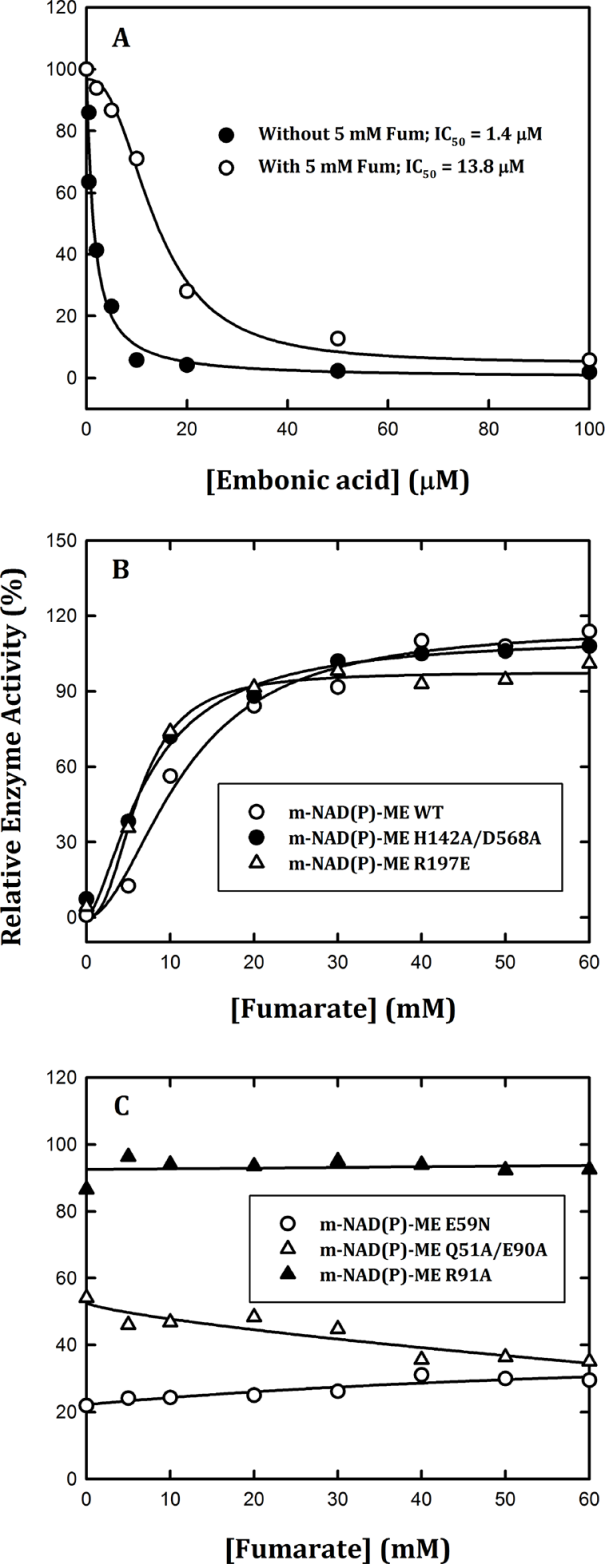
Effects of fumarate on the reversible inhibition of human m-NAD(P)-ME enzyme activity by EA The assay mixture contained 40 mM malate, 10 mM MgCl_2_, 2 mM NAD^+^ and 50 mM Tris-HCl (pH 7.5). **A.** m-NAD(P)-ME was pre-incubated with 5 mM fumarate and then assayed with various concentrations of EA; **B.** m-NAD(P)-ME WT (open circles), H142A/D568A (closed circles), and R197E (open triangles) were pre-incubated with 40 μM EA and then assayed with various concentrations of fumarate; **C.** E59N (open circles), Q51A/E90A (closed circles), and R91A (open triangles) were pre-incubated with 40 μM EA and then assayed with various concentrations of fumarate.

Whether the EA-inhibited m-NAD(P)-ME activity could be restored by fumarate was further investigated. First, we found that fumarate had a protective effect on m-NAD(P)-ME activity (Figure 3A). In the presence of fumarate (5 mM), the IC_50_ value increased to 13.8 ± 1.0 μM, 10-fold greater than in the absence of fumarate (1.4 ± 0.4 μM). Moreover, we examined the fumarate effect on the EA-inhibited enzyme activity of WT and mutants. The enzyme was pre-incubated with 40 μM EA to inhibit the enzyme activity to the maximal level. For m-NAD(P)-ME WT, H142A/D568A, and R197E, which were sensitive to EA inhibition (Figure 2, 2B and 2C), the EA-inhibited enzyme activity could be reactivated by fumarate to more than 90% of the original enzyme activity (Figure 3B). In contrast, for m-NAD(P)-ME Q51A/E90A, E59N, and R91A, which were less sensitive or insensitive to EA inhibition (Figure 2, 2A and 2D), the EA-inhibited enzyme activity could not be restored by fumarate (Figure 3C). These data again suggested that EA is bound to the fumarate binding site or at the dimer interface near the site.

### Inhibition patterns of embonic acid (EA)

**Figure 4:**
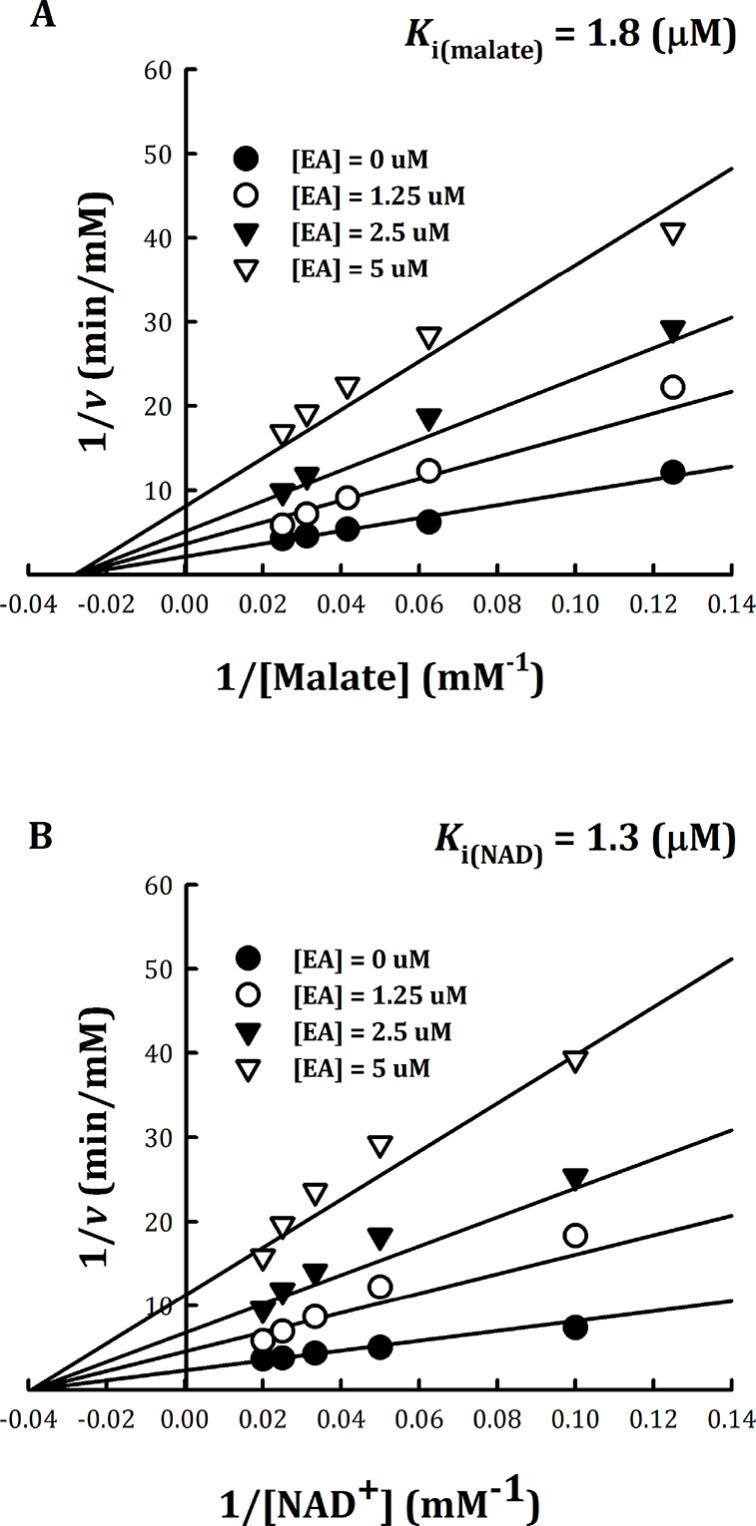
Non-competitive inhibition of human m-NAD(P)-ME by embonic acid (EA) Human m-NAD(P)-ME activities were measured using different concentrations of **A.** malate or **B.** NAD^+^ with various concentrations of EA; the concentrations of EA were 0, 1.25, 2.5 and 5 μM from the bottom to top.

The inhibition pattern of EA with respect to the substrate L-malate and NAD^+^ were examined, and the inhibition constants, *K*_i(malate)_ and *K*_i(NAD)_, were determined (Figure 4). Kinetic analysis indicated that m-NAD(P)-ME displayed a non-competitive inhibition pattern with EA with respect to either L-malate or NAD^+^ (Figure 4). The *K*_i(malate)_ and *K*_i(NAD)_ values of EA for human m-NAD(P)-ME were 1.8 ± 0.2 μM and 1.3 ± 0.1 μM, respectively, similar to the IC_50_ value (1.4 ± 0.4 μM). According to the mutagenesis analysis and kinetic studies, as well as the ability of fumerate to reverse the enzyme inhibition by EA, the putative binding site of EA was proposed to be at the fumarate binding site or at the dimer interface near the site. The non-competitive inhibition pattern, which demonstrated that the binding site of EA was distinct from the active site of the enzyme, coincided with this postulation.

### Binding characteristics of embonic acid (EA) for m-NAD(P)-ME

**Figure 5:**
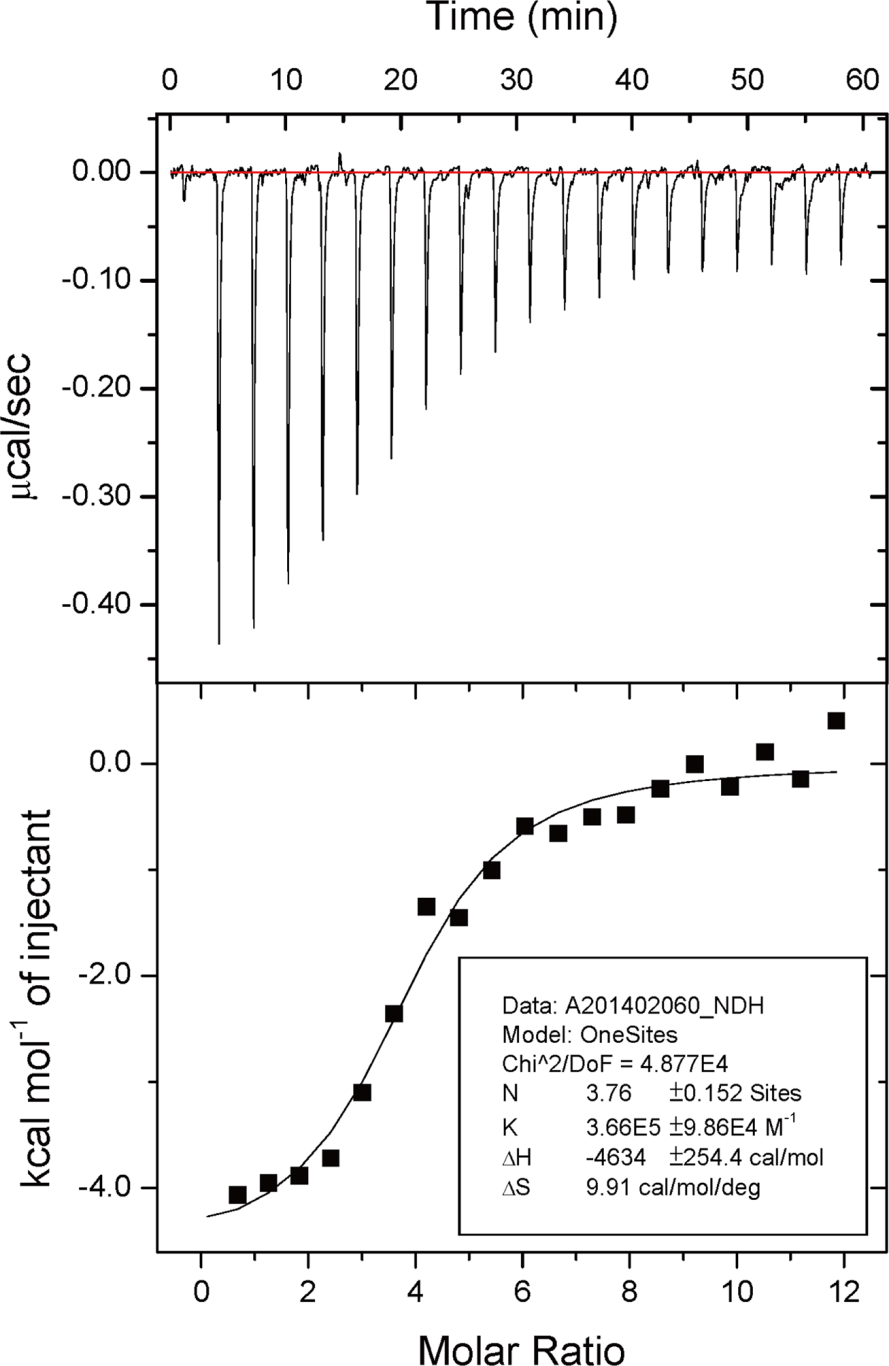
Isothermal titration calorimetry data of embonic acid (EA) against m-NAD(P)-ME Human m-NAD(P)-ME (70 μM, 30 mM HEPES, pH 7.4) was titrated with EA (10 mM) at 298K. The upper panel shows the raw data curve, and the lower panel shows the fitted integrated ITC data curve. The data were fitted with the “ONESites” model of the MicroCal (Northampton, MA) version of ORIGIN 7.0.

We further investigated the stoichiometry and dissociation constant (*K*_d_) of EA for m-NAD(P)-ME WT and mutants using isothermal titration calorimetry (iTC). The microcalorimetric titration plots of m-NAD(P)-ME WT and mutants during the EA titration were shown in Figure 5 and Supplementary Figure S1. m–NAD(P)-ME is a homotetramer with four identical active centers, fumarate binding sites and exo sites; the iTC data were well fitted with a “OneSites” model, indicating no cooperative binding of EA to the respective sites. The best fit of the dissociation constant of EA for m–NAD(P)-ME and their relative thermodynamic parameters are shown in Table 2. The binding of EA to m–NAD(P)-ME WT was an exothermic reaction (Figure 5). The *K*_d_ value of EA toward the WT enzyme was 2.7 ± 0.7 μM with an averaged *N* value of 3.8 ± 0.2 sites; the Δ*G*, Δ*H* and *T*Δ*S* values were −7.6, −4.6, and 3.0 kcal/mol, respectively (Table 2). This fit revealed that the average number of EA molecules bound to m–NAD(P)-ME was approximately 3.8, very close to the theoretical value of 4, and the binding of EA to the enzyme was energy-favorable.

**Table 2:** Thermodynamic parameters determined for the interaction of embonic acid with human m-NAD(P)-ME

Characteristics	m-NAD(P)-ME	^a^Average *N* (sites)	^b^*K*_d_ (μM)	^c^Δ*G* (kcal/mol)	^d^Δ*H* (kcal/mol)	^f^TΔ*S* (kcal/mol)	^e^Δ*S* (kcal/mol/deg)
	WT	3.8 ± 0.2	2.7 ± 0.7	−7.6	−4.6 ± 0.3	3.0	9.9
*Tetramer interface mutant*	H142A/D568A	1.4 ± 0.03	0.4 ± 0.1	−8.8	−12.3 ± 0.4	−3.5	−11.7
*Exo site mutant (at tetramer interface)*	R197E	3.4 ± 0.1	1.0 ± 0.2	−8.2	−5.1 ± 0.1	3.1	10.5
*Dimer interface mutant*	Q51A/E90A	1.8 ± 0.1	1.1 ± 0.4	−8.1	4.7 ± 0.4	12.8	42.8
*Fumarate site mutant (at dimer interface)*	E59N	2.0 ± 0.2	3.5 ± 1.1	−7.4	−1.7 ± 0.2	5.8	19.4
	R67A	1.9 ± 0.2	4.0 ± 0.7	−7.3	4.0 ± 0.2	11.4	38.1
	R91A	3.7 ± 0.4	13.1 ± 3.8	−6.7	−1.7 ± 0.3	5.0	16.5

a Average *N* (sites) signifies the stoichiometry of embonic acid in a tetrameric malic enzyme at equilibrium.

b *K*_d_ (μM) represents the dissociation constant of embonic acid against m-NAD(P)-ME, which is reciprocal of *K*_A_ (association constant).

c Δ*G* (kcal/mol)

d Δ*H* (kcal/mol) and

e Δ*S* (kcal/mol/deg) represent the free energy change during the binding process.

f T was fixed at 298K.

The binding of EA to the mutant enzymes was also examined (Supplementary Figure S1). The tetramer interface mutant, H142A/D568A, displayed a *K*_d_ value of 0.4 ± 0.1 μM with an average *N* value of 1.4 ± 0.03 sites, and the Δ*G*, Δ*H* and *T*Δ*S* values were −8.8, −12.3, and −3.5 kcal/mol, respectively (Table 2, Supplementary Figure S1A). Although the binding stoichiometry of H142A/D568A (N = 1.4 sites) is smaller than that of WT (N = 3.8 sites), the binding affinity (*K*_d_ = 0.4 μM) is also smaller than that of WT (*K*_d_ = 2.7 μM) and the free energy change (Δ*G* = −8.8 kcal/mol) is more negative than that of WT (Δ*G* = −7.6 kcal/mol). These results might be the reason that the IC_50_ of H142A/D568A is similar to that of WT. It is interesting that the entropy change of H142A/D568A (Δ*S* = −11.7 kcal/mol/deg) is negative, indicating that the binding of EA to H142A/D568A reduced the entropy, which implied that this binding induced an unfavorable conformational change. Additionally, this enzyme is the only mutant in which the entropy was reduced after EA binding.

The exo site mutant, R197E, displayed a *K*_d_ value of 1.0 ± 0.2 μM with an average *N* value of 3.4 ± 0.1 sites, and the Δ*G*, Δ*H* and *T*Δ*S* values were −8.2, −5.1, and 3.1 kcal/mol, respectively (Table 2; Supplementary Figure S1B). This mutant is most similar to WT in terms of EA binding characteristics. The binding stoichiometry of R197E (N = 3.4 sites) is comparable to that of WT (N = 3.8 sites), although the binding affinity (*K*_d_ = 1.0 μM) is a little smaller than that of WT (*K*_d_ = 2.7 μM). The free energy, enthalpy and entropy changes (Δ*G,* Δ*H* and Δ*S*) of R197E are equivalent those of WT. This result indicated that the disruption of the exo site of the enzyme did not significantly affect the binding characteristics of EA for m-NAD(P)-ME.

The dimer interface mutant, Q51A/E90A, displayed a *K*_d_ value of 1.1 ± 0.4 μM with an average *N* value of 1.8 ± 0.1 sites, and the Δ*G*, Δ*H* and *T*Δ*S* values were −8.1, 4.7, and 12.8 kcal/mol, respectively (Table 2; Supplementary Figure S1C). The binding stoichiometry of Q51A/E90A (N = 1.8 sites) is less than that of WT (N = 3.8 sites), and the binding affinity (*K*_d_ = 1.1 μM) is also smaller than that of WT (*K*_d_ = 2.7 μM). Although the free energy change of Q51A/E90A (Δ*G* = −8.1 kcal/mol) is comparable to WT (Δ*G* = −7.6 kcal/mol), the enthalpy change was switched to positive and the entropy change increased greatly (Δ*H* = 4.7 kcal/mol and Δ*S* = 42.8 kcal/mol/deg) compared to WT (Δ*H* = −4.6 kcal/mol and Δ*S* = 9.9 kcal/mol/deg). The IC_50_ value of Q51A/E90 is approximately 30-fold greater than that of WT. These results indicated that the disruption of the dimer interface of the enzyme significantly affected the binding characteristics of EA for m-NAD(P)-ME.

The binding characteristics of the fumarate site mutants E59N, R67A, and R91A for EA were examined. The Glu59 residue is an indirect binding ligand of fumarate. The E59N mutant displayed a *K*_d_ value of 3.5 ± 1.1 μM with an average *N* value of 2.0 ± 0.2 sites, and the Δ*G*, Δ*H* and *T*Δ*S* values were −7.4, −1.7, and 5.8 kcal/mol, respectively (Table 2; Supplementary Figure S1D). The binding stoichiometry of E59N (N = 2.0 sites) was less than that of WT (N = 3.8 sites), and the binding affinity of this mutant (*K*_d_ = 3.5 μM) was slightly decreased compared to WT (*K*_d_ = 2.7 μM). The free energy, enthalpy and entropy changes (Δ*G,* Δ*H* and Δ*S*) of E59N maintained a similar trend compared to those of WT. The Arg67 and Arg91 residues are direct binding ligands of fumarate. The R67A mutant displayed a *K*_d_ value of 4.0 ± 0.7 μM with an average *N* value of 1.9 ± 0.2 sites, and the Δ*G*, Δ*H* and *T*Δ*S* values were −7.3, 4.0, and 11.4 kcal/mol, respectively (Table 2; Supplementary Figure S1E). The binding stoichiometry of R67A (N = 1.9 sites) was also less than that of WT (N = 3.8 sites), and the binding affinity (*K*_d_ = 4.0 μM) was slightly decreased compared to WT (*K*_d_ = 2.7 μM). Although the free energy change of R67A (Δ*G* = −7.3 kcal/mol) is comparable to WT (Δ*G* = −7.6 kcal/mol), the enthalpy change was switched to positive and the entropy change was increased greatly (Δ*H* = 4.0 kcal/mol and Δ*S* = 38.1 kcal/mol/deg) compared to that of WT (Δ*H* = −4.6 kcal/mol and Δ*S* = 9.9 kcal/mol/deg). The R91A mutant displayed a large *K*_d_ value of 13.1 ± 3.8 μM with an average *N* value of 3.7 ± 0.4 sites, and the Δ*G*, Δ*H* and *T*Δ*S* values were −6.7, −1.7, and 5.0 kcal/mol, respectively (Table 2; Supplementary Figure S1F). The binding stoichiometry of R91A (N = 3.7 sites) was comparable to that of WT (N = 3.8 sites), but the binding affinity (*K*_d_ = 13.1 μM) was significantly decreased compared to WT (*K*_d_ = 2.7 μM). The free energy, enthalpy and entropy changes of R91A (Δ*G,* Δ*H* and Δ*S*) maintained a similar trend compared to those of WT. To briefly summarize the iTC data of these mutants of m-NAD(P)-ME, these fumarate site mutants displayed larger *K*_d_ values, especially for R91A, indicating that the abolishment of the fumarate binding ligand of the enzyme truly has an effect on the binding affinity of EA to m-NAD(P)-ME.

Based on the IC_50_ and *K*_d_ values in combination with the enzyme activity reversibility and inhibition pattern studies, we suggest that EA is preferentially bound to the fumarate binding site or at the dimer interface near the site. First, the IC_50_ values of fumarate site mutants (E59N, R67A, R91A, and K57S/E59N/K73E/D102S) were greater than 75 μM, which was much greater than that of WT. The IC_50_ value of the dimer-interface mutant Q51A/E90A also showed an IC_50_ value significantly larger than that of WT, indicating that disruption of the dimer interface that had an effect on the binding of EA. Second, although the average number of EA bound to the WT and mutant enzymes was diverse, the *K*_d_ values of fumarate site mutants increased a certain amount. This observation is especially true for R91A, which had a similar *N* value but larger IC_50_ and *K*_d_ values compared to WT. We had tried to resolve the structure of the EA-enzyme complex; however, the enzyme complexed with EA failed to co-crystallize. Based on the present *in vitro* studies, it is clear that the binding site of EA is not at the active site or at the exo site. EA was most likely bound to the fumarate binding site or the dimer interface near the site.

### Quaternary structure of m-NAD(P)-ME in the presence of embonic acid (EA)

As previously determined, the dissociation of m-NAD(P)-ME tetramers into dimers reduces the enzyme's activity [31]. Here, we examined the effect of EA on the enzyme's quaternary structure (Supplementary Figure S2). The sedimentation distribution plot of human WT m-NAD(P)-ME displayed a dimer-tetramer equilibrium in solution. When the enzyme was incubated with EA (2 and 5 mM), it also exhibited tetrameric and dimeric quaternary structures similar to the pattern of the enzyme without EA (Supplementary Figure S2). These data clearly indicated that the binding of EA to the enzyme did not cause any change in the enzyme's quaternary structure and that the inhibitory effect of EA on the enzyme is not associated with the enzyme's dissociation.

### Embonic acid (EA) inhibited the growth of H1299 cancer cells through the suppression of *in vivo* ME2 activity

**Figure 6:**
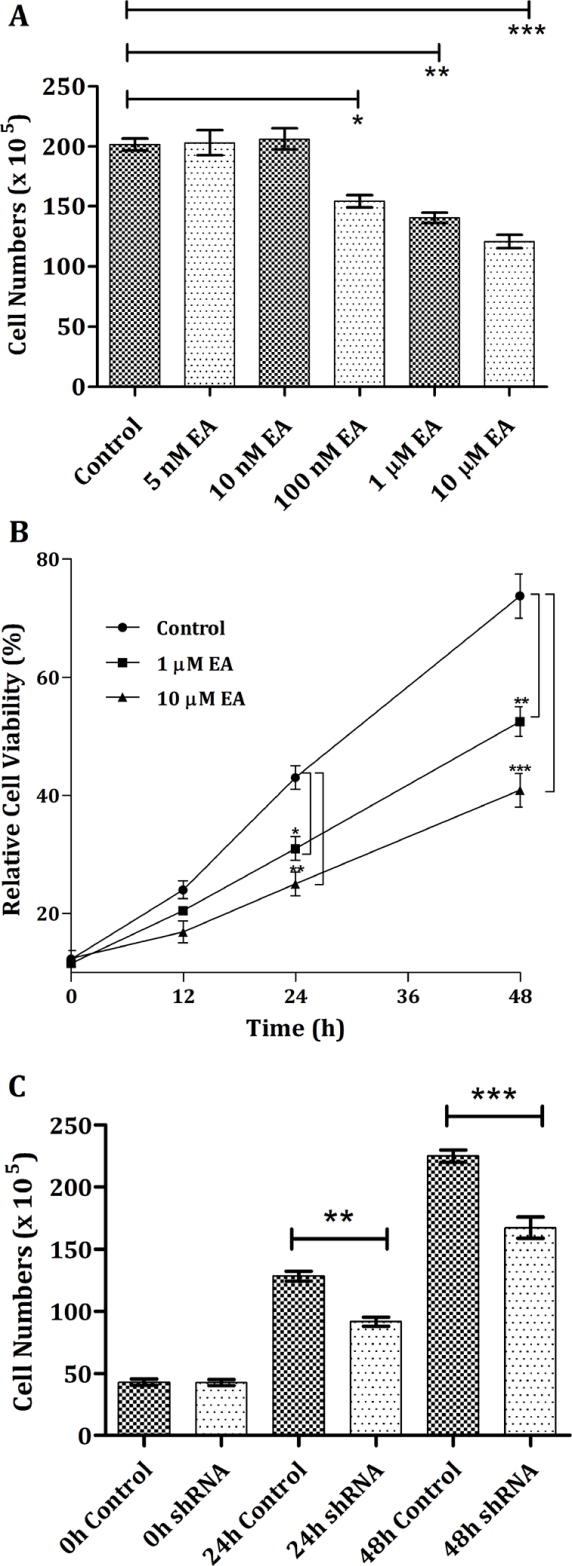
Cell growth inhibition of H1299 cancer cells through the addition of embonic acid (EA) **A.** The dosage performance was examined by treating H1299 cells with vehicle (control), 5, 10, 100 nM, 1 or 10 μM EA for 24 h. **B.** To determine the time dependence, cells were treated with 0, (control), 1 or 10 μM EA for 0, 12, 24, 36 or 48 h. **C.** H1299 cells were pretreated with shLuc (control) or ME2 shRNA for 0, 24 or 48 h. Total cells were harvested, and cell numbers were calculated and determined at indicated time and dose points. *P < 0.05, **P < 0.01 and ***P < 0.001.

Certain types of cancer cell lines overexpresses m-NAD(P)-ME. In the current study, the human non-small cell lung carcinoma cell line H1299 was utilized to investigate the effect of EA on cancer cells. First, the dose effect of EA on H1299 cells was examined. After 24 h, the H1299 cell number did not decrease after treatment with 5 nM or 10 nM EA (Figure 6A). The cell number notably decreased after treatment with 100 nM, 1 μM, or 10 μM EA (10%, one-fold and 10-fold concentrations of the *in vitro* IC_50_ value, respectively); the cell numbers decreased 23%, 30% and 40%, respectively, compared with the control (Figure 6A). Second, the time-course effects of EA on H1299 cells were examined. Without EA treatment, the growth of H1299 cells increased in a time-dependent manner (Figure 6B, closed circles). However, within 48 h, treatment with 1 μM EA inhibited H1299 cell growth, and this phenomenon was more pronounced with 10 μM EA (Figure 6B, closed squares and triangles, respectively). Cell growth inhibition was also observed in m-NAD(P)-ME-knockdown H1299 cells. The cell number decreased after treatment with shRNA, which can silence ME2 expression; the cell viability was approximately 72% and 68% of the control after 24 h or 48 h, respectively (Figure 6C). These data indicated that both treatment with EA and knockdown of m-NAD(P)-ME by shRNA had similar effects on the inhibition of H1299 cell growth.

The protein and mRNA levels of m-NAD(P)-ME in H1299 cells were also examined. Briefly, EA treatment did not suppress the transcriptional or translational levels of m-NAD(P)-ME. The protein expression and mRNA synthesis of m-NAD(P)-ME in H1299 cells was not influenced by EA (Supplementary Figures S3 and S4, respectively). Based on the aforementioned results, we suggest that the inhibition of H1299 cell growth by EA occurs through suppression of *in vivo* m-NAD(P)-ME activity.

### Embonic acid (EA)-induced senescence of H1299 cancer cells

**Figure 7:**
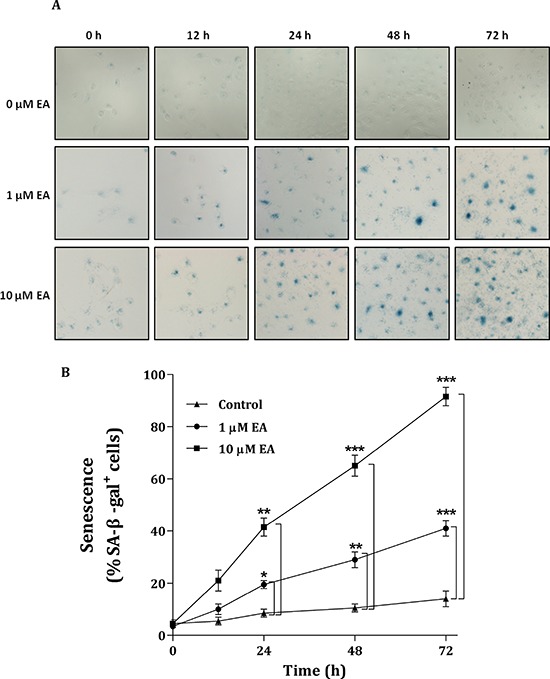
Senescence of H1299 cancer cells induced by embonic acid (EA) The dose and time dependencies were determined by treating H1299 cells with different concentrations of EA (0, 1 and 10 μM) for different amounts of time (0, 12, 24, 48 and 72 h), respectively. **A.** Cells were stained with 5-bromo-4-chloro-3-indolyl β-D-galactoside (X-Gal) and detected using the senescence-associated β-galactosidase (SA-β-gal) assay. The image was analyzed by light microscope. **B.** The percentages of SA-β-gal^+^ cells were calculated by the number of positively cells per 1,000 cells counted in ten random fields. *P < 0.05, **P < 0.01 and ***P < 0.001.

ME2 has been reported to down-regulate enzyme-induced cellular senescence [16]. In our study, we found that EA can induce the senescence of H1299 cancer cells. EA treatment caused a profound increase in the expression of senescence-associated β-galactosidase by H1299 cells (Figure 7A). In the absence of EA, the senescent signal, SA-β-gal^+^ H1299 cells, was not present (Figure 7A, upper panels). SA-β-gal^+^ H1299 cells were present after a 12-h EA treatment. After treatment with 1 μM EA, the number of SA-β-gal^+^ H1299 cells increased in a time-dependent manner (Figure 7A, middle panels). Within 72 h, the EA-induced H1299 cell senescence was more prominent after treatment with 10 μM EA (Figure 7A, lower panels). During this time-course experiment, the amount of senescence after treatment with 10 μM EA was 2-fold greater than that after treatment with 1 μM EA (Figure 7B). These data indicated that the EA-induced senescence of H1299 cells occurred through the depletion of m-NAD(P)-ME enzyme activity. However, down-regulation of the enzyme-induced cellular senescence did not occur through p53 because H1299 is a p53-null cancer cell line. Therefore, the EA-induced senescence of H1299 cells may occur directly through the inhibition of ME2 or a p53-independent pathway.

## CONCLUSION

This work reports a natural compound, embonic acid, which can specifically inhibit m-NAD(P)-ME, an allosteric isoform of human malic enzymes. This inhibitor exhibited excellent efficacy with a very small IC_50_ value of approximately 1 μM, which is smaller than that of some preclinical drugs. Mutagenesis analysis revealed that the putative binding site of EA on the human m-NAD(P)-ME may be located at the fumarate binding site or at the dimer interface near the site. Therefore, EA is thought to be an allosteric inhibitor specific to m-NAD(P)-ME. Furthermore, this inhibitor can suppress the growth of H1299 cancer cells and induce cellular senescence. Because of its low IC_50_ value and anti-cancer activity, we believe that EA could be a good candidate for the development of anticancer drugs, and these results should greatly advance our understanding in this field.

## MATERIALS AND METHODS

### Chemicals

Embonic acid (EA) was purchased from ChromaDEX (Irvine, CA, USA).

### Expression and purification of recombinant malic enzymes

The procedures for the expression and purification of human m-NAD(P)-ME and c-NADP-ME have been described in earlier reports [9, 30]. Briefly, m-NAD(P)-ME was subcloned into the pRH281 expression vector and transformed into the BL21 strain of *Escherichia coli*; the expression of m-NAD(P)-ME was controlled by an inducible *trp* promoter system. c-NADP-ME was subcloned into the pET21b vector, which is ampicillin-resistant and contains a His6· Tag sequence for purification. The c-NADP-ME-containing pET21b vector was transformed into the BL21(DE3) strain of *E. coli*, and its expression was controlled by an inducible T7 promoter system.

m-NAD(P)-ME was purified using column chromatography; the lysates were first passed through a DEAE-sepharose column (Amersham Biosciences, Uppsala, Sweden), followed by an ATP-agarose column (Sigma, St Louis, MO, USA). The m-NAD(P)-ME was eluted from the resin with elution buffer containing 4 mM NAD^+^ and 30 mM Tris-HCl (pH 7.4). c-NADP-ME was purified using Ni-NTA sepharose (Sigma, St Louis, MO, USA). The c-NADP-ME was eluted from the resin with elution buffer containing 250 mM imidazole, 500 mM sodium chloride, 2 mM β-mercaptoethanol and 30 mM Tris-HCl, pH 7.4. After purification, the enzymes were dialyzed and concentrated using a centrifugal filter device (Amicon Ultra-15, Millipore, Billerica, MA, USA) with a molecular weight cutoff of 30 kDa in a buffer containing 30 mM Tris-HCl (pH 7.4) and 2 mM β-mercaptoethanol.

### Site-directed mutagenesis

Site-directed mutagenesis of human m-NAD(P)-ME was accomplished using the QuikChange^TM^ kit (Stratagene, La Jolla, CA, USA). Most mutants in this paper were created previously [26, 32, 37]. Briefly, a high fidelity *Pfu* DNA polymerase was used in the PCR reactions; the specific primers including the desired mutation sites were approximately 25–45 nucleotides in length, which was necessary for the specific binding of the template DNA. After 16–18 temperature cycles, staggered nicks were created in the mutated plasmids. The PCR product was then treated with DpnI to digest the wild-type m-NAD(P)-ME templates. Lastly, the nicked DNA with the desired mutations was transformed into the XL-1 *E. coli* strain, and their DNA sequences were confirmed by sequencing.

### Enzyme kinetic analysis

The malic enzyme reaction was assayed in a reaction buffer containing 50 mM Tris-HCl (pH 7.4) with saturated concentrations of L-malate, NAD^+^ or NADP^+^ and MgCl_2_, with various concentrations of EA in a total volume of 1 ml at 30°C. The absorbance at 340 nm was continuously monitored in a UV/VIS spectrophotometer Lambda 25 (Perkin Elmer, MA, USA). In this process, one unit of enzyme was defined as the amount of enzyme that produces 1 μmol of NAD(P)H per min under the assay conditions. An absorption coefficient of 6.22 cm^−1^mM^−1^ for NAD(P)H was used in the calculations.

The inhibition experiments were conducted with a series of EA concentrations in a buffer containing 50 mM Tris-HCl (pH 7.4), 40 mM malate (pH 7.4), 10 mM MgCl_2_ and 2 mM NAD^+^ (pH 7.4). The *K*_i_ determination of the enzyme was assayed with a reaction buffer consisting of 50 mM Tris-HCl (pH 7.4) and 10 mM MgCl_2_; the reactions were supplemented with a series of EA concentrations close to its IC_50_ value and a series of NAD^+^ or malate (pH 7.4) concentrations close to its *K*_m_ value. The total dataset was globally fitted using the following equation, which describes a non-competitive inhibition pattern:

$$v=\frac{V_{\text{max}}\times [\text{S}]}{\left(K_{\text{m}}+[\text{S}])\times (1+\frac{\left[\text{I}\right]}{K_{\text{i}}}\right)}$$

where *v* is the observed initial velocity, *V*_max_ is the maximum rate of the reaction, *K*_m_ is the Michaelis constant for NAD^+^ or malate, and *K*_i_ is the inhibition constant for EA.

### Isothermal titration calorimetry (iTC)

The enzyme solutions (70 μM) were loaded into an iTC_200_ isothermal titration calorimeter (Microcal, Inc.), which has an active cell volume of 280 μl. The solution was titrated with 20 injections of a 10 mM EA solution at 3-minute intervals using a 2-μl titration syringe. The stirring speed was set to 1000 rpm. The experiments were conducted at a constant temperature of approximately 25°C. The ligand solutions were prepared in the same buffer (HEPES, pH 7.4) as the protein solutions. The titration experiments were repeated three times. The raw data were corrected for dilution effects, and the concentration was normalized prior to data analysis using the “ONESites” model of the MicroCal version of ORIGIN 7.0. During the fitting process, *K*_A_ (association constant) and Δ*H* were allowed to float.

### Quaternary structure analysis of malic enzyme by analytical ultracentrifugation

Sedimentation velocity experiments of m-NAD(P)-ME were carried out using a Beckman Optima XL-A analytical ultracentrifuge. The samples (380 μL) and the buffer solutions (400 μL) were individually loaded into the double sector centerpiece and assembled in a Beckman An-50 Ti rotor. The experiments were performed at 20°C with a rotor speed of 42,000 rpm for 3.5 to 4 hours. The protein samples were continuously monitored by UV absorbance at 280 nm with a time interval of 480 seconds and a step size of 0.002 cm. Multiple scans of the sedimentation velocity data were collected and analyzed using the software SEDFIT 9.4c [38, 39]. All size distributions were solved with a confidence level of *p* = 0.95, a best-fitted average anhydrous frictional ratio (*f/f_0_*) and a resolution N of 200 sedimentation coefficients between 0.1 and 20.0 S.

### Cell viability of H1299 cells during embonic acid (EA) or ME2 shRNA treatment

H1299 cells were grown in 90% Dulbecco's Modified Eagle Medium (DMEM) with 10% fetal bovine serum (FBS) (Gibco BRL, USA) at a temperature of 37°C in a humidified 5% CO_2_ environment. Cells were seeded in 6-well cell culture dishes in triplicate at a density of 1 × 10^5^ cells/ml medium containing 10% FBS and treated with EA or siRNA at the indicated dosage. The cells were then counted, and cell numbers were determined at the indicated time points. The control shLuc and ME2 shRNA were purchased from the Nature RNAi Core Facility, Taipei, Taiwan (NRC). The shME2 was made using pLKO 005 (TRC2 vector). The target shRNA sequence for ME2 is 5′-GCACGGCTGAAGAAGCATATA-3′, and the target shRNA sequence for luciferase is 5′-GCGGTTGCCAAGAGGTTCCAT-3′.

### Senescence of H1299 cells during embonic acid (EA) treatment

Dimri et al. (1995) first reported the senescence-associated β-galactosidase (SA-β-gal) assay, demonstrating that its expression distinguishes senescent cells from proliferating and quiescent cells [40]. The SA-β-gal activity in cultured H1299 cells treated with 0, 1 or 10 μM EA for 0, 12, 24, 48 or 72 h was determined to demonstrate EA-induced cellular senescence. Cells were washed in PBS, fixed for 4 min at room temperature in approximately 2 to 3% formaldehyde or 0.2% glutaraldehyde, washed, and then incubated at 37°C under no C0_2_ conditions with fresh SA-β-Gal stain solution. Cells were stained with 5-bromo-4-chloro-3-indolyl β-D-galactoside (X-Gal) for 2 to 16 h and detected using the senescence-associated β-galactosidase (SA-β-gal) assay. The percentages of SA-β-gal^+^ cells were calculated by the number of positively stained cells per 1,000 cells counted in random fields.

### Immunoblotting analysis of human m-NAD(P)-ME in H1299 cancer cells during embonic acid (EA) treatment

To extract the total cell proteins, H1299 cells were lysed in cold lysis buffer (10% v/v glycerol, 1% v/v Triton X-100, 1 mM sodium orthovanadate, 1 mM EGTA, 10 mM NaF, 1 mM sodium pyrophosphate, 20 mM Tris, pH 7.9, 100 μM β-glycerophosphate, 137 mM NaCl, 5 mM EDTA, 1 mM PMSF, 10 μg/ml aprotinin, and 10 μg/ml leupeptin), and the cell lysates were subsequently homogenized and centrifuged. The supernatants were boiled in loading buffer, and an aliquot containing 100 μg of protein was separated by SDS-PAGE. After blotting, the PVDF membranes were incubated with anti-human m-NAD(P)-ME (ME2) (Sigma, St Louis, MO) and anti-β-actin antibodies (Santa Cruz) for 6 h, and membranes were then incubated for 1 h with the second antibody labeled with horseradish peroxidase. The antigen-antibody complexes were visualized using enhanced chemiluminescence (Amersham Pharmacia Biotech, USA).

### Reverse transcription polymerase chain reaction (RT-PCR)

Total RNA was isolated from H1299 cells treated with shME2 or EA for 12 h by using a total RNA reagent (MDBio, Inc., Taiwan). One microgram of RNA was reverse transcribed into cDNA using MMLV reverse transcriptase (Epicentre Biotechnologies, USA). PCR was performed in the linear range (by 25 cycles) with primers specific for ME2 and actin. The primer sequences used to amplify the target genes were as follows: ME2 forward primer, 5′-AGAGCTAGCCCAAGGGAGAC-3′; ME2 reverse primer, 5′-TCAACACGTCTACCCCAACA-3′; actin forward primer, 5′-CCCTATCATCTTTGCCCTGA-3′, and reverse primer, 5′-GGAAGCCAGGTTGTGTTTGT-3′.

## SUPPLEMENTARY FIGURES


