# Supplementary material for: A small-molecule inhibitor suppresses the tumor-associated mitochondrial NAD(P)+-dependent malic enzyme (ME2) and induces cellular senescence
Source: Oncotarget. 2015 May 19;6(24):20084–98. doi: 10.18632/oncotarget.3907 (PMC4652989; doi:10.18632/oncotarget.3907)
Supplement: Supplementary file 1 [file oncotarget-06-20084-s001.pdf]

## SUPPLEMENTARY FIGURES

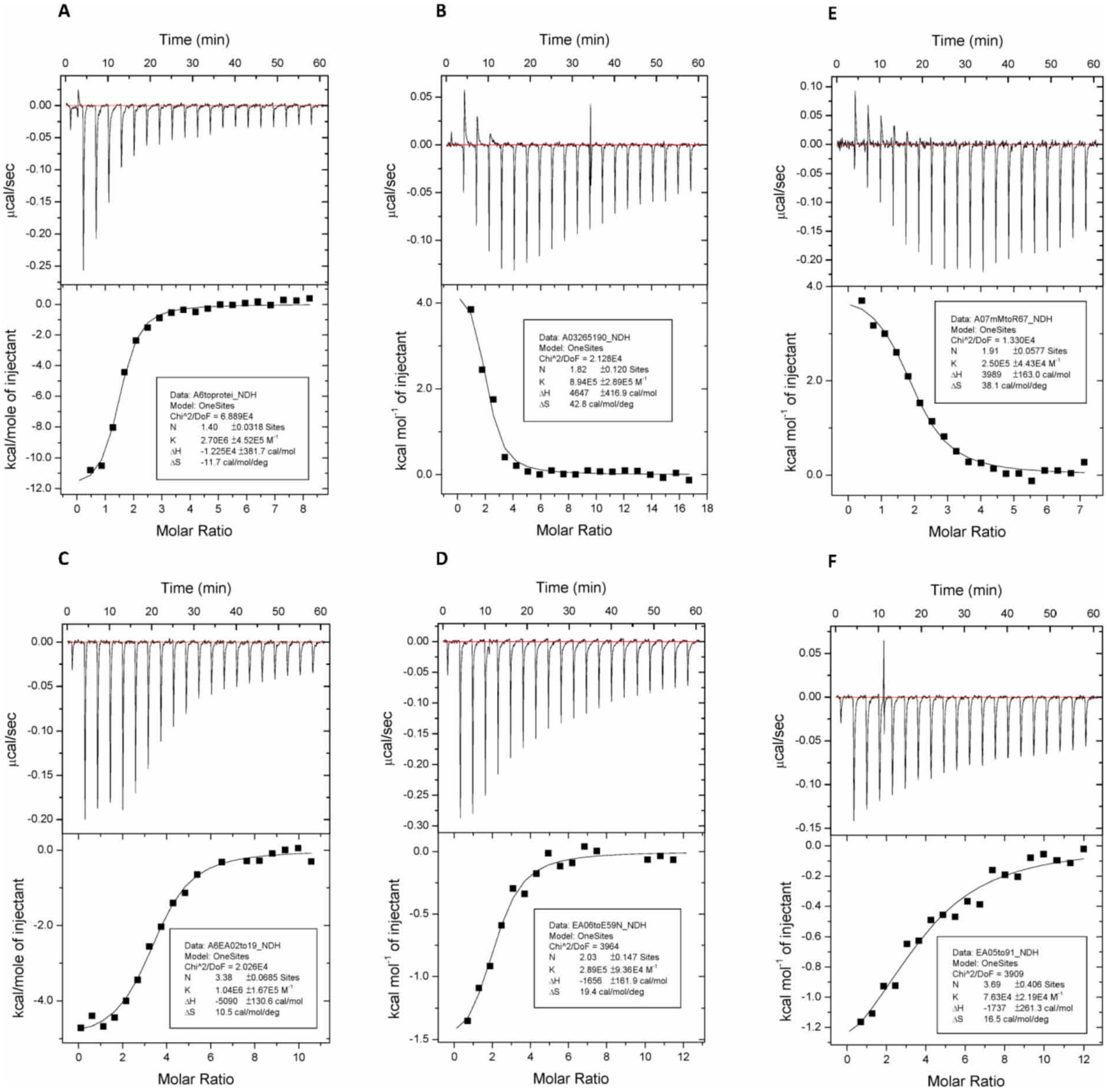


**Supplementary Figure S1: Isothermal titration calorimetry data of embonic acid (EA) against m-NAD(P)-ME mutants.** **A.** H142A/D568A tetramer interface mutant; **B.** R197E exo site mutant; **C.** Q51A/E90A dimer interface mutant; **D.** E59N, **E.** R67A, and **F.** R91A fumarate site mutants. The upper panel shows the raw data curve, and the lower panel shows the fitted integrated ITC data curve. The data were fitted with the "ONESites" model of the MicroCal (Northampton, MA) version of ORIGIN 7.0.

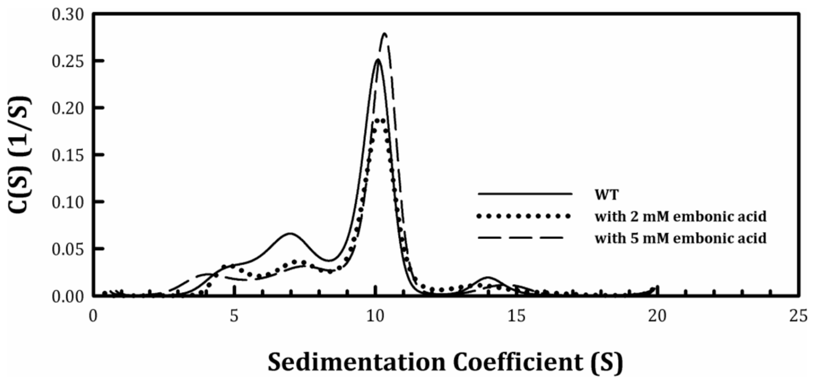


**Supplementary Figure S2: Sedimentation plot of m-NAD(P)-ME in the presence of embonic acid (EA).** The enzymes (0.5 mg/ml) in 30 mM Tris-HCl (pH 7.4) were run in an analytical ultracentrifuge at 20°C. Solid line: m-NAD(P)-ME without EA; dotted line: m-NAD(P)-ME with 2 mM EA; dashed line: m-NAD(P)-ME with 5 mM EA.

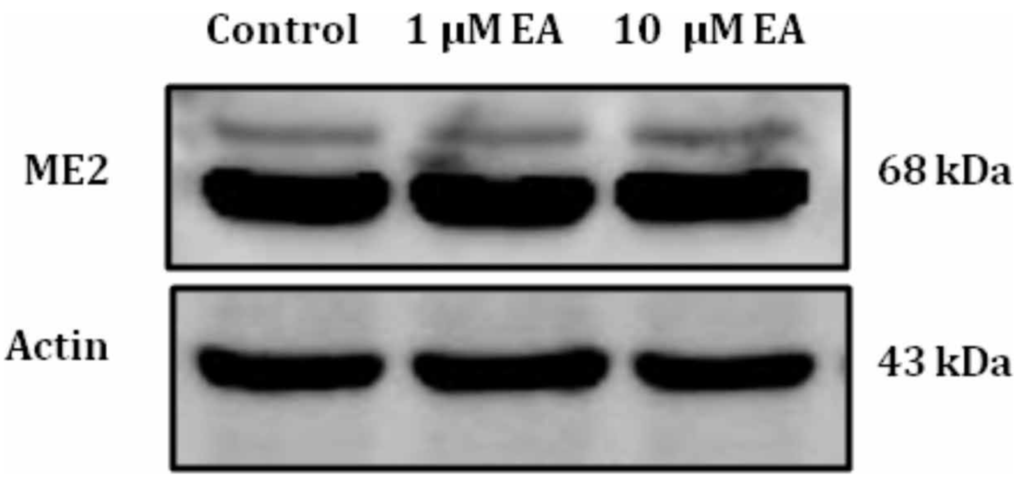


**Supplementary Figure S3: Protein levels of m-NAD(P)-ME in H1299 cancer cells in the presence of embonic acid (EA).** The protein expression levels of human m-NAD(P)-ME (68 kDa) and actin (43 kDa) in H1299 lung cancer cells, which were treated with various concentrations of EA for within 24 h, were detected by Western blot.

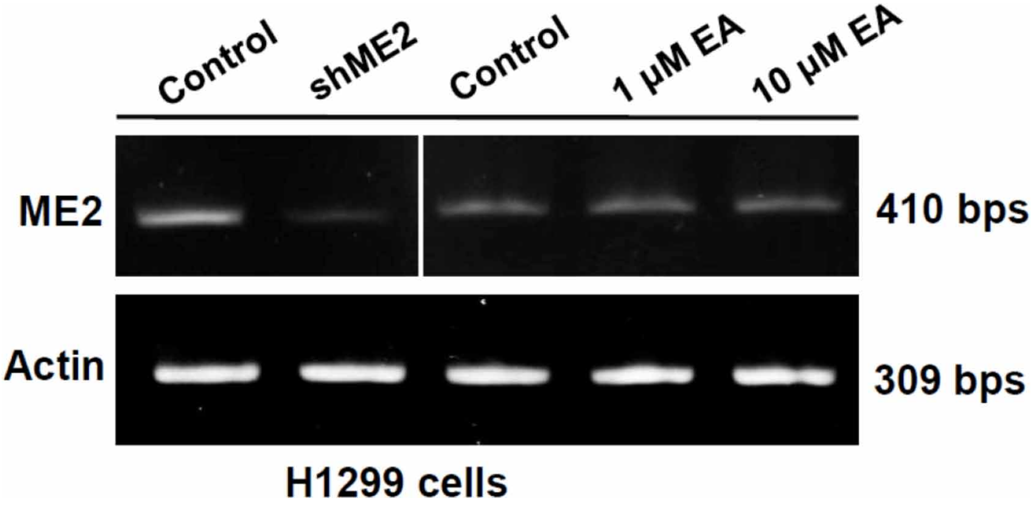


**Supplementary Figure S4: The mRNA levels of m-NAD(P)-ME in H1299 cancer cells after treatment with shME2 or embonic acid (EA).** The mRNA expression levels of human m-NAD(P)-ME (410 bps) and actin (309 bps) in H1299 lung cancer cells, which were treated with various concentrations of EA for within 12 h, were detected by RT-PCR.
